# Frequency and accuracy of intraoperative bone margin sampling for T4a cancers of the head and neck at the QEII Health Sciences Centre: a retrospective chart review

**DOI:** 10.1186/s40463-022-00609-2

**Published:** 2023-01-31

**Authors:** Anna-Claire Lamport, Colin A. MacKay, Martin J. Bullock, S. Mark Taylor, Jonathan R. Trites, Martin Corsten, Matthew H. Rigby

**Affiliations:** 1 Faculty of Medicine, Dalhousie University, Halifax, NS Canada; 2 Division of Otolaryngology–Head and Neck Surgery, Dalhousie University and Queen Elizabeth II Health Sciences Centre, Halifax, NS Canada; 3 Department of Pathology, Dalhousie University and Queen Elizabeth II Health Sciences Centre, Halifax, NS Canada

**Keywords:** Bone margins, Stage T4a, Intraoperative margin sampling

## Abstract

**Background:**

Stage T4a cancers are associated with a 5-year survival of 21.6–59.0%. Adequate resection of these tumors is a critical factor in maximizing survival. Tumors invading bone pose a unique challenge to intraoperative bone margin assessment. Due to processing limitations, there had been no formal standardized protocol for intraoperative bone sampling at the QEII Health Sciences Centre. These resections often involve extensive reconstruction, making salvage surgery difficult if positive margins are detected post-surgically. The purpose of this study was to assess the accuracy and frequency of intraoperative bone margin assessment during the study period and to determine survival and recurrence rates associated with positive final bone margins.

**Methods:**

A retrospective chart review was conducted including patients with stage T4a head and neck cancer involving bone that underwent primary surgical resection in Nova Scotia between 2009 and 2019. Eligible patients were identified through the Cancer Care Nova Scotia registry. Exclusion criteria included patients with stage T4a tumors involving bone that did not receive primary surgical treatment with curative intent and patients with stage T4a tumors that did not invade bone.

**Results:**

Of 67 patients included, 50 were amenable to intraoperative bone margin sampling while 18 had intraoperative sampling. Four patients had positive intraoperative margins and one had final positive bone margins. The incidence of final bone margin positivity was 7.5%. Median survival following surgery was 4.56 years for patients with final negative bone margins (n = 62) and 3.98 years for patients with positive final bone margins (n = 5). All patients with final positive bone margins received adjuvant radiation therapy. Of patients with negative final bone margins, 16.1% received no adjuvant therapy, 61.3% received adjuvant radiation therapy and 21.0% received adjuvant chemoradiation therapy.

**Conclusion:**

Intraoperative bone margin sampling occurred in 26.8% of all cases and 36.0% of amenable cases. Median survival of patients with positive final bone margins was 0.58 years lower than those with negative final bone margins, although this difference did not reach statistical significance. This will provide baseline data for comparison of the standardized intraoperative bone margin sampling protocol implemented at the QEII Health Sciences Centre.

**Graphical Abstract:**

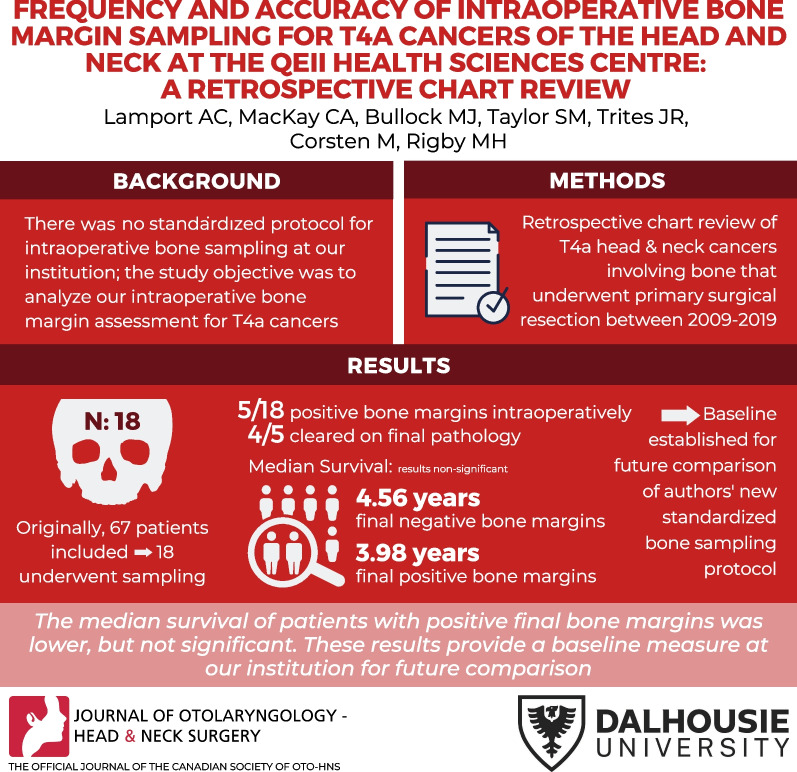

## Background

Head and neck cancers pose a significant threat to a patient’s quality of life due to the integral nature of the structures involved. Stage T4a head and neck cancer represents advanced disease characterized by invasion into bone, nerves, deep tongue muscles or the skin of the face. These tumors can be resected surgically, but are associated with worse outcomes. Five-year overall survival has been reported to be 45–59% [1, 2] for stage T4a oral cavity cancers and 21.6% [3] for stage T4a oropharyngeal cancers. Adjuvant therapy is often used in patients with a high risk of recurrence following surgery, such as those with positive margins or involvement of multiple lymph nodes. Addition of chemotherapy to primary surgical and/or radiation therapy has been shown to reduce mortality by 11% [4]. Additionally, combined chemoradiation therapy has been shown to prolong recurrence-free survival relative to radiation therapy alone [5]. However, combined chemoradiation therapy is associated with nearly double the risk of severe debilitating side effects such as nausea, mucositis, hematologic toxicity and treatment-related death [4, 5]. Measures that reduce the requirement for adjuvant therapy following primary surgical treatment would help to prevent the incidence of these debilitating side effects.

Head and neck tumors involving bone pose a unique challenge to the intraoperative assessment of margin status. Intraoperative margin assessment allows a surgeon to revise the tumor resection if positive margins are detected. For analysis of dense cortical bone, such as that of the mandible, decalcification must occur before margins can be assessed through histopathology. Adequate decalcification currently requires 12–24 hours [6], meaning standard protocols cannot provide information regarding margin status during surgery. Salvage surgery is generally impractical if positive margins are detected after surgery because residual disease is often buried beneath a complex bony reconstruction. Diagnostic imaging modalities such as CT, MRI and bone scintigraphy are lacking in their ability to accurately estimate degree of bone invasion [7] and imaging can be complicated by artefact after complex reconstructions. There is therefore a need for a standard processing method to assess bone margins rapidly.

A number of methods for rapid assessment of bone margins have been proposed. This includes the use of an osteotome to produce very thin samples that can be analyzed through standard frozen sectioning without decalcification [9] and the use of a bone drill to produce fine bone fragments that require only 30 min of decalcification [6]. Another promising technique is outlined in a study by Bilodeau and Chiosea [10], which included 27 patients that underwent segmental mandibulectomy for squamous cell carcinoma. This study assessed the utility of intraoperative sampling of bone marrow and inferior alveolar nerve as a marker for mandibular bone margins, which yielded a sensitivity of 50% and specificity of 100%. Another study that assessed intraoperative mandibular bone marrow sampling alone reported a sensitivity of 88.9% and a specificity of 100% [8]. There is currently no standardized method that is widely accepted for the assessment of intraoperative bone margins in head and neck cancers. When assessment of bone margins intraoperatively has occurred at the QEII Health Science Centre, it has occurred through sampling of bone marrow, small bone fragments and/or inferior alveolar nerve.

The objectives of this study are two-fold; the first is to assess the rate of final positive bone margins following resection of head and neck tumors invading bone at the QEII Health Science Centre and resulting oncologic outcomes. This will allow for comparison of our centre’s outcomes to those reported in the literature. The second objective is to assess the current intraoperative bone margin sampling practices. This will provide baseline data for comparison of the standardized intraoperative bone margin sampling protocol implemented at the QEII Health Sciences Centre.

## Methods

### Ethics

Ethics approval was received from the Nova Scotia Health Authority Research Ethics Board (NSHA REB #1020700).

### Patients

Patients eligible for the study included those who have undergone primary surgical resection of a head and neck cancer with bone involvement at the QEII Health Science Centre in Halifax, Nova Scotia, Canada between January 1^st^, 2009 and January 1^st^, 2019. Surgery must have been performed by a surgeon from the Division of Otolaryngology – Head and Neck Surgery. Exclusion criteria included having undergone resection of a stage T4a tumor without bone infiltration. This study involved patients with resections of the oral cavity, oropharynx and maxilla.

### Intraoperative bone margin sampling methods

Intraoperative bone margin sampling occurred through histopathological analysis of mandibular bone marrow, inferior alveolar nerve and mandibular bone fragments that were small enough to be assessed without decalcification. Bone marrow and small bony fragments were collected through curettage of the marrow space. Intraoperative margin samples were frozen and sectioned with a microtome. Sections were then stained with hematoxylin and eosin (H&E) and assessed by a pathologist.

### Data collection

Data collected for the study from patient charts included patient demographics, tumor-specific data, intra-operative surgical details, pathology reports, incidence of recurrence and survival data. Due to the observational nature of the study, researchers were not blinded.

### Statistical analysis

Data analysis was performed using Prism 8. Recurrence-free survival (RFS) and overall survival (OS) were analyzed using Kaplan–Meier survival curves. Kaplan–Meier curves were compared using a log rank test.

## Results

### Patients

A total of 67 patients were eligible for inclusion in the study. Patient demographics are presented in Table 1. The ratio of men to women who underwent resection of a stage T4a tumor involving bone during this timeframe was approximately 2:1.

**Table 1:** Demographics of patients with negative and positive bone margins on final surgical pathology

	Negative bone margins	Positive bone margins
Number of patients	62	5
Age, mean (range)	62.7 (36–88)	75.6 (67–83)
*Sex, no. (%)*		
M	40 (65)	4 (80)
F	22 (35)	1 (20)
*Node status*		
0	27	0
1	9	1
2a	1	0
2b	14	0
2c	6	3
3	1	0
Not assessed	1	0
Not reported	3	1
Recurrence from previous head and neck cancer	8	2
Final soft tissue margins positive	6	1
*Bone resected*		
Segmental mandibulectomy	33	2
Marginal mandibulectomy	5	1
Partial maxillectomy	6	1
Infrastructure maxillectomy	2	0
Segmental mandibulectomy + partial maxillectomy	5	0
Marginal mandibulectomy + partial maxillectomy	2	0
Hyoid	4	0
Segmental mandibulectomy + styloid process	1	0
Partial maxillectomy + pterygoid plate	1	0
Partial maxillectomy + nasal bone	1	0
Partial maxillectomy + ethmoid bone + sphenoid bone + orbit	0	1
Segmental mandibulectomy + hyoid	1	0
Radical maxillectomy + frontal bone + sphenoid bone	1	0

### Final surgical margins

Of the 67 included patients, 62 (92.5%) had negative bone margins and 5 (7.5%) had positive margins at the time of final pathology. Of the 5 patients with positive final bone margins, two had a segmental mandibulectomy, one had a marginal mandibulectomy, one had a partial maxillectomy and one had a partial maxillectomy with resection of the sphenoid, ethmoid and orbital bones. Seven patients (10.4%) had positive soft tissue margins on final pathology, one of which also had final positive bone margins. In one patient with positive final bone margins, soft tissue margins could not be determined. Fifty patients (74.6%) had a resection involving mandible, while the remainder involved resection of maxilla, hyoid and/or other facial bones only (Table 1).

### Recurrence-free survival

**Fig. 1:**
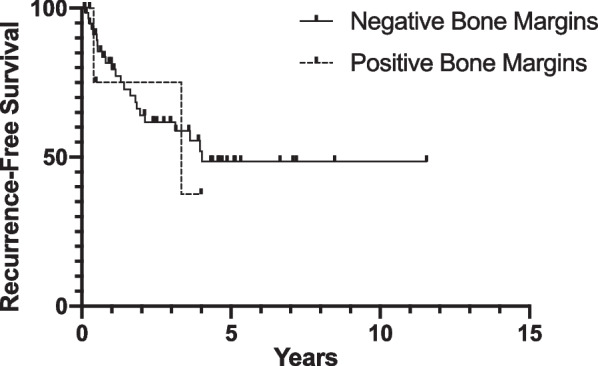
Percent recurrence-free survival of patients with negative vs. positive bone margins on final surgical pathology measured by date of first recurrence (negative margins n = 62, positive margins n = 5)

The median RFS was 4.03 years for patients with final negative bone margins and 3.34 years for patients with final positive bone margins (p = 0.66; Fig. 1). The 5-year RFS was 48.6% for patients with final negative bone margins and estimated to be 37.5% for patients with final positive bone margins (Fig. 1).

### Overall survival

**Fig. 2:**
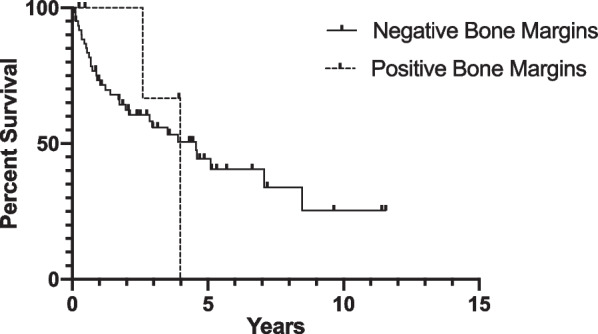
Percent survival of patients with final negative or positive bone margins (negative margins n = 62, positive margins n = 5)

The median OS was 4.56 years for patients with final negative bone margins and 3.98 years for patients with final positive bone margins (p = 0.96; Fig. 2). Five-year OS for patients with final negative bone margins was 44.3%, whereas no patients with final positive bone margins survived five years after surgery. Disease specific survival could not be determined due to a lack of consistent access to cause of death.

### Intraoperative bone margin sampling

Of the 67 patients in the study, 50 involved resection of mandible and were therefore amenable to intraoperative bone margin sampling based on previous institution sampling practices. A total of 18 patients underwent intraoperative bone margin sampling. Intraoperative bone margin sampling occurred through analysis of curreted tissue of the medullary space including mandibular bone marrow and/or small bone fragments (13/18), and the inferior alveolar nerve (IAN) when present at resection site (7/18). Five patiets did not have sufficient bone marrow harvested and underwent analsysis of the IAN alone. Fourteen patients had negative intraoperative bone margins, all of which had negative final bone margins. Four patients had positive intraoperative bone margins, three of which had negative final bone margins. Two patients with positive intraoperative bone marrow sampling had further resection of mandible and negative final bone margins. Another patient had intraoperative sampling of the inferior alveolar nerve after the mandible had been resected, which was positive. There was further resection of soft tissue with positive final soft tissue margins and negative final bone margins. The remaining patient with positive intraoperative bone margins had a re-resection of bone intraoperatively, however the bone margins remained positive on final pathology. Sensitivity and specificity could not be determined as pathologists did not routinely assess the bone margins of initial specimens if re-resections were performed after a positive intraoperative bone margin was identified.

### Adjuvant therapy

Of the patients with final negative bone margins, 10 (16.1%) received no adjuvant therapy, 38 (61.3%) received radiation therapy and 13 (21.0%) received chemoradiation (Table 2). For one (1.6%) patient in this group, there was no access to information on adjuvant therapy given. All 5 (100%) patients with final positive bone margins received adjuvant radiation therapy alone. Two of the patients did not receive chemotherapy in addition to radiation due to patient preference and one did not receive chemotherapy due to age and functional status. It is unknown why the remaining two patients did not receive adjuvant chemotherapy.

**Table 2:** Adjuvant therapy administered in patients with negative and positive final bone margins, absolute numbers (percent)

	Negative bone margins	Positive bone margins
Radiation alone	38 (61.3)	5 (100)
Chemotherapy alone	0	0
Chemoradiation	13 (21.0)	0
No adjuvant therapy	10 (16.1)	0
No data	1 (1.6)	0

## Discussion

This study assessed the outcomes of patients with stage T4a head and neck cancer involving bone following primary surgical resection as well as the effectiveness of intraoperative bone margin sampling at the QEII Health Sciences Centre between 2009 and 2019. During the study period, 7.5% of stage T4a cancers involving bone had positive final bone margins following primary surgical treatment. This is within the range reported in the literature, and compares favourably with the upper end of the range, as positivity rates of 21.3% have previously been reported [10, 11].

The positive soft tissue margin rate of 10.4% in this patient cohort is higher than our institutional rate for patients with T1-T4 cancers of the oral cavity and oropharynx that did not involve bone [12]. While this is intuitive given the aggressive nature of tumours that invade bone, it is an area for consideration moving forward about how to better assess the soft tissue margins in these cases.

A study by Smits et al. [11] reported an overall 5-year survival of 23.0% for patients with final positive bone margins and 35.3% for patients with final negative bone margins. Our study demonstrates a higher overall 5-year survival of 44.3% for patients with negative final bone margins. No patients with final positive bone margins were alive at 5 years. Positive margins would typically be an indication for the addition of chemotherapy, however none of the five patients with positive margins received chemotherapy in addition to radiation treatment. This was due to patient preference, age and functional status, and the reasons unknown in two cases. This may have been one factor contributing to the poor survival outcomes associated with positive bone margins. The median survival of those with positive final bone margins was 0.58 years less than of those with negative final bone margins, but this difference was not statistically significant. Only five patients had positive final bone margins in our study, making meaningful statistical analysis difficult.

Intraoperative bone margin sampling occurred in 36.0% of cases involving resection of mandible, or 26.8% of all cases in this study. All instances of intraoperative bone margin sampling at our institution occurred in cases involving resection of mandible, due to the ease of access to bone marrow. The remainder of resection involved maxilla, hyoid and other cranial bones that are typically not amenable to bone marrow sampling. Of those with intraoperative sampling, 4 out of 18 (22.2%) were positive and one remained positive on final pathology despite further resection. These metrics will serve as a baseline comparator for a recently implemented standardized intraoperative sampling protocol at the QEII Health Sciences Centre. During the study period, four out of five patients with positive final bone margins did not have intraoperative bone margin sampling. Therefore, the goal of this protocol is to increase the incidence of intraoperative bone margin sampling in cases involving resection of mandible. Introduction of a standardized protocol will allow for better detection of positive bone margins, which is a known predictor of worse outcomes.

## Conclusions

Positive margin status is a known predictor of worse outcomes in head and neck cancer. In our study, those with positive final bone margins displayed worse morbidity and mortality outcomes compared to those with negative final margins, although this difference did not reach statistical significance. Only one-third of patients that underwent resection of mandible had intraoperative bone margin sampling, which identifies patients likely to have residual disease after resection. This study is relevant for establishing baseline intraoperative bone margin sampling rates and disease outcomes at our centre. This will be used as a benchmark for comparing the recently implemented standardized sampling protocol. The aim is to increase the rate of intraoperative sampling of amenable resection sites to greater than 90% and ultimately reduce margin positivity rates and improve disease outcomes.

## Data Availability

Deidentified data used in analysis is available upon reasonable request.

## Abbreviations

OS: Overall survival
RFS: Recurrence-free survival
